# Healthcare Providers’ Perspectives on Telemedicine for NCD Management During and After COVID-19 in India: A Qualitative Study

**DOI:** 10.3390/ijerph23020203

**Published:** 2026-02-05

**Authors:** Mohd Shannawaz, Isha Rathi, Nikita Shah, Shazina Saeed, Amrish Chandra

**Affiliations:** 1 Amity Institute of Public Health & Hospital Administration, Amity University, Noida 201301, India; 2 Lexgin IP LLP, Noida 201301, India

**Keywords:** telemedicine, non-communicable diseases, COVID-19, healthcare providers, India

## Abstract

**Highlights:**

**Public health relevance—How does this work relate to a public health issue?**

- Telemedicine played a crucial role in sustaining non-communicable disease (NCD) care during the COVID-19 pandemic in India.
- Healthcare providers’ experiences provide insights into the real-world use of telemedicine in resource-limited settings such as those with poor internet, staff shortages, limited time, or low digital skills.

**Public health significance—Why is this work of significance to public health?**

- This study provides qualitative evidence on the system-level, technological, and clinical challenges affecting the long-term integration of telemedicine into routine NCD care.
- Provider-recommended strategies, including workflow restructuring, digital literacy strengthening, and hybrid care models, align with national digital health goals.

**Public health implications—What are the key implications or messages for practitioners, policy makers and/or researchers in public health?**

- Addressing infrastructure gaps, improving digital competencies, and incorporating video-enabled and hybrid models are essential for sustainable telemedicine adoption.
- Future digital health policies and research should incorporate provider insights and follow patients over time, using mixed-method approaches to ensure equitable and resilient NCD care delivery.

**Abstract:**

Non-communicable diseases (NCDs) remain a major contributor to global morbidity and mortality, with India bearing a substantial share of this burden. The COVID-19 pandemic disrupted routine clinical care and accelerated the transition to telemedicine for chronic disease management. This study explores healthcare providers’ perspectives on the use of telemedicine for NCD care during and after the pandemic, including its perceived benefits, limitations, and future relevance within the broader digital health landscape. Semi-structured interviews were conducted with 33 purposively selected healthcare providers with experience in telemedicine for NCD care between February and June 2025, audio-recorded, transcribed verbatim, and thematically analyzed using NVivo 15. Providers reported that telemedicine supported continuity of care, reduced the need for in-person visits, facilitated medication adherence, and offered reassurance for patients during periods of restricted mobility. However, limitations were identified, including the inability to conduct physical examinations, connectivity challenges, and low digital literacy, particularly among older adults. While overall satisfaction with telemedicine was positive, participants emphasized the need for strengthened digital infrastructure, greater patient awareness, and simplified platforms to improve usability. The findings underscore telemedicine’s continued relevance for NCD management beyond the pandemic, contingent on improved accessibility and system integration.

## 1. Introduction

Globally, non-communicable diseases (NCDs) pose a significant health concern and have emerged as a leading cause of morbidity and mortality, particularly in low- and middle-income countries (LMICs) [1,2]. According to the World Health Organization (WHO), in 2021, NCDs caused approximately 43 million deaths, representing 75% of all global fatalities unrelated to the pandemic, with 73% of these occurring in LMICs [3]. Evidence shows that South Asia, notably India and Nepal, is experiencing a notable epidemiological transition, with non-communicable diseases accounting for a growing proportion of the disease burden [4,5,6].

Self-care practices and lifestyle changes are essential components for managing non-communicable diseases, along with regular medical check-ups to monitor health conditions [7,8,9]. The management of non-communicable diseases in large populations is challenging, as these conditions require ongoing monitoring and sustained long-term care, while regular medical checkups can be time-intensive and burdensome for some patients [8,10,11]. Healthcare providers can use telemedicine to deliver remote consultations and monitoring, improving access to care in remote and underserved areas and enhancing temporal accessibility while reducing reliance on in-person visits [12].

Amid the COVID-19 pandemic, telemedicine emerged as a globally embraced tool for overcoming the limitations of traditional in-person consultations [13]. According to the WHO, digital health interventions, including mobile phones, online portals, and other digital tools, can play a vital role in achieving universal health coverage and delivering quality care [14]. India’s public health strategies are increasingly integrating digital healthcare solutions. In 2021, the government launched the Ayushman Bharat Digital Mission (ABDM) to develop a unified national digital health ecosystem [15].

India’s doctor-to-patient ratio stands at approximately 1:1500, falling short of the WHO-recommended standard of 1:1000, with rural areas facing an even greater shortfall at nearly 1:2500, resulting in unequal access to healthcare services across the country [16]. Telemedicine emerges as a beacon of hope by making services more accessible and cost-effective, especially for underserved populations [17].

This qualitative study aimed to explore the challenges and barriers associated with telemedicine use, assess its perceived benefits in managing NCDs in India, particularly during and after the COVID-19 pandemic, and understand healthcare providers’ perspectives on its future potential.

## 2. Methodology

### 2.1. Study Design

This qualitative interview study was conducted in accordance with the Consolidated Criteria for Reporting Qualitative Research (COREQ); the checklist can be found in Supplementary Table S1. In this study, a descriptive qualitative approach was used to obtain a deep and detailed understanding of the data. The qualitative research was conducted through interviews to effectively explore the experiences and perspectives of healthcare professionals. Prior to the interview, oral informed consent was obtained from all participants after being fully informed about the study’s objectives and the use of the collected data. Participation was voluntary, confidentiality was maintained, and participants were allowed to withdraw from the study at any point.

### 2.2. Sampling and Recruitment

Participants were selected through purposive sampling to capture maximum variation in socio-demographic characteristics, including age, gender, expertise, and years of work experience. This sampling approach was aligned with the exploratory aims of the qualitative study. Healthcare providers with prior experience of telemedicine were included to ensure that participants could meaningfully reflect on the practical benefits, challenges, and limitations of technology-supported care in real-world clinical settings. Accordingly, the sampling strategy was designed to capture in-depth experiential insights.

Eligible participants included healthcare professionals with experience in managing NCDs who had prior exposure to telemedicine for NCD-related consultation or follow-up during or after the COVID-19 pandemic; policymakers engaged in health promotion or technology-enabled care; and researchers or academicians specializing in public health, digital health, or NCD prevention. Healthcare professionals with no prior exposure to telemedicine for NCD care were excluded. Participants were recruited from government hospitals, private hospitals and clinics, and academic institutions. They were contacted by the researchers NS and IR via email and direct messages; after the initial contact, there were no dropouts. The sample size was guided by the principles of code saturation and meaning saturation, whereby data collection continued until no additional themes or insights emerged [18]. A total of 33 interviews was conducted to capture diverse insights, with data saturation confirmed through team discussions (IR, NS, and MS) once no new themes emerged.

### 2.3. Data Collection

Data was collected through semi-structured, in-depth interviews with participants, guided by an interview guide. The interview guide was pilot-tested with a senior healthcare provider to assess the clarity, relevance, and flow of the questions. Feedback from the pilot interview was used to refine the wording and sequencing of the questions, and the pilot interview was not included in the final analysis. The complete interview guide is available in Supplementary Material Table S2. Interviews were carried out from February 2025 to June 2025, and oral informed consent was obtained from all participants before the interview commenced. Each interview was audio-recorded, lasted 20 to 25 min, and was transcribed verbatim, with no field notes documented during the process.

### 2.4. Interview Procedure

Based on participants’ preferences, interviews were conducted either face-to-face or over the phone, at a time convenient for them. We used a semi-structured interview guide with open-ended questions to encourage participants to provide detailed accounts of their experience. All interviews were conducted by a single interviewer, IR, a female Ph.D. scholar with experience in public health research and conducting in-depth interviews. IR had no prior close relationship with the participants.

### 2.5. Bias Mitigation Strategies

Several strategies were employed to minimize potential biases during data collection and analysis. The research team-maintained reflexivity by using a reflexive journal to critically examine how their own perspectives could potentially influence the data. Data source triangulation was achieved by comparing insights across participants from diverse professional backgrounds. Peer debriefing was undertaken, wherein the research team regularly discussed emerging interpretations with colleagues not directly involved in the data collection; this process facilitated the identification and challenge of underlying assumptions and contributed to a more balanced and comprehensive analysis. The adoption of these strategies enhanced the quality and trustworthiness of the study.

### 2.6. Data Analysis

In this qualitative study, data analysis was conducted in the chronological order of the interviews and followed a concurrent design, with analysis ongoing throughout data collection. First, a preliminary transcript was generated using Microsoft Word Online’s transcription feature, an AI-powered tool that utilizes speech recognition technology to convert audio into text, enabling the efficient transcription of audio recordings. Then the transcript was manually reviewed and corrected by IR and NS by cross-checking with the original audio recordings. All identifiable information was removed to ensure anonymity, and the final transcripts were then imported into NVivo 15 for analysis by IR.

The transcripts were analyzed using a thematic analysis approach for the identification of patterns and themes within the qualitative data. This approach ensured the systematic inclusion of the perspectives and viewpoints of the participants. IR and NS independently coded the interviews to develop a code structure, which was then reviewed and finalized by the research team (IR, NS, and MS). IR grouped the codes into categories by constant comparison and applying theoretical sensitivity and used participant quotes to support and highlight the identified themes. The final themes were reviewed by all team members, with any discrepancies resolved through discussions. Transcripts and key findings were subsequently shared with participants for their feedback.

## 3. Results

A total of *n* = 33 participants, ranging in age from 28 to 73 years and comprising 17 females and 16 males, agreed to participate in the study. They were selected from diverse healthcare settings, including government and private hospitals, primary health centres (PHCs), private clinics, and academic institutions. Participants included healthcare providers (HCPs) such as doctors, pharmacists, and AYUSH (**A**yurveda, **Y**oga & **N**aturopathy, **U**nani, **S**iddha, and **H**omeopathy) professionals, as well as medical and public health academics. A detailed description is provided in Table 1.

### 3.1. Thematic Analysis

Thematic analysis was conducted using an inductive–deductive approach, and the coding tree was structured around the study’s core areas of interest. We identified three major themes, 10 subthemes, and a total of 44 codes. The detailed coding tree is presented in Supplementary Material Table S3.

### 3.2. Theme 1: Perceived Benefits and Satisfaction with Telemedicine

#### 3.2.1. Access, Affordability, and Time–Cost Efficiency

Telemedicine emerged as a valuable tool for improving healthcare accessibility, particularly in rural and remote areas where physical access to healthcare facilities is limited. Participants also perceived telemedicine as a convenient, time- and cost-efficient alternative that helped patients avoid wage loss and the additional expenses associated with travel for in-person consultations.

“*For middle and lower socioeconomic statuses, daily wages matter a lot when coming to the healthcare system; taking consultation and one day’s wages is curtailed, is cut down. So, regarding this point and also the difficulty in coming, getting a consultation, and wasting a whole day is not affordable for all people. So, for follow-up services, telemedicine can be a good additional point apart from traditional consultation*” (P07).

#### 3.2.2. Continuity of Care During the COVID-19

Most participants reported that telemedicine played a critical role in ensuring continuity of care during COVID-19 lockdowns while simultaneously reducing the burden on healthcare facilities. It was perceived as particularly effective for routine follow-ups in the management of chronic conditions.

#### 3.2.3. Psychological Reassurance and Perceived Support

Beyond clinical care, telemedicine was perceived to provide psychological reassurance. Participants noted that the ability to communicate remotely with healthcare providers helped alleviate anxiety and offered patients a sense of support, especially during the pandemic when in-person visits were restricted.

“*Seeing the covid situation. It had more pros than cons; health was the top priority, and being able to talk to a doctor during such a crisis wasn’t just about managing the disease, it also gave patients a lot of psychological relief. Just knowing they could reach out and get guidance really helped ease their worries*” (P021).

#### 3.2.4. Provider Satisfaction with Telemedicine

Provider satisfaction was assessed using a self-reported rating scale ranging from 1 to 10. Overall, responses reflected a favorable perception of telemedicine. Nearly half of the participants (48.5%) reported high satisfaction (scores of 8–10), citing convenience, time-saving potential, and usefulness during both the COVID-19 pandemic and the post-COVID period. Approximately 39.4% reported moderate satisfaction (scores of 6–7), acknowledging telemedicine’s benefits while noting limitations such as the absence of physical examinations and face-to-face interaction. A smaller proportion (12.1%) rated their satisfaction below 6, expressing concerns related to clinical limitations, doctor–patient rapport, and the need for stronger system support.

“*Telemedicine has been extremely beneficial, be it COVID or non-COVID times. It provides ease of consultation, so I would rate it 9 out of 10*” (P17).

### 3.3. Theme 2: Challenges in Telemedicine Use

#### 3.3.1. Clinical Limitations

Participants reported a wide range of challenges associated with the use of telemedicine for NCD management during the pandemic. A major concern voiced by most providers was the inability to conduct physical examinations remotely.

“*We couldn’t see the patients. We couldn’t touch the patients; direct physical examination was not possible, right? So, it is the major challenge*” (P11).

Furthermore, it was noted that while telemedicine was effective for follow-ups, it posed challenges for new cases.

“*It is very difficult to initially diagnose the patient, but for the follow-up, it is very good, but for the initial check on examination and for history taking, it is very difficult in telemedicine*” (P08).

#### 3.3.2. Technological and Infrastructure Barriers

In addition to clinical limitations, infrastructural barriers were repeatedly emphasized, especially in rural and remote regions. Poor internet connectivity, power outages, and limited access to suitable devices are frequent disruptions to teleconsultations. As one participant mentioned, even when connectivity was stable on the provider’s end, patients frequently struggled with unstable networks or outdated technology, making effective communication difficult.

“*First of all, the awareness is lacking among people; 80% of people don’t know. Also, to avail this service, you need to have strong internet or infrastructure. In rural areas we still lack internet; there is a lot to be done in these areas*” (P032).

Additionally, the absence of visual interaction limited providers’ ability to observe facial expressions and nonverbal cues.

“*We do have telemedicine; the visual is not available here. Visual things face-to-face—if it is available, it will be good; it will be better to consult them. With their reaction facial expressions, we can catch some problems*” (P11).

#### 3.3.3. Digital Literacy and Heterogenous Awareness

Digital literacy was a significant challenge, especially among elderly patients who constitute a significant portion of those with NCDs. Many were unfamiliar with smartphones or video consultation platforms and often required assistance from family members to share reports or communicate with healthcare providers.

“*So, most of the patients, like if you see, among a hundred patients, 60 or 70 will be more than 60 years of age. So, accessing mobile phones and accessing video conferencing apps is difficult for them*” (P06).

Participants also highlighted that the absence of face-to-face interaction made it difficult to establish rapport and communicate effectively. The lack of visual cues and limited time often led to misunderstandings, particularly among elderly patients who preferred in-person consultations for more personal and comprehensive discussions.

“*From the patient side, especially elderly patients prefer face-to-face visits because they want to discuss more things apart from their medical condition, and online consultations often feel less personal and are of short duration, like 2–3 min, and can leave patients unsatisfied*” (P14).

Moreover, lack of awareness among lower-income groups posed a significant barrier to effective telemedicine utilization.

“*As far as India is concerned, we are basically dealing with the poor class; most of them are not aware of telemedicine*” (P02).

#### 3.3.4. System-Level and Scheduling Constraints

Participants reported challenges in aligning consultation times with patients’ availability, especially when patients were occupied with work during standard clinic hours.

“*We are working for a certain period of time; the patient is also working. So, they will be wanting a time maybe before or after that certain time, like in the morning or after evening, so technically that is not possible for us*” (P028).

#### 3.3.5. Data Safety, Privacy, and Regulatory Concerns

Participants expressed varied perceptions regarding data privacy in telemedicine. Urban and higher socioeconomic groups expressed concerns about data misuse and cyber threats, whereas individuals from rural or lower-income backgrounds were generally less aware or unconcerned. Moreover, patients tend to trust their doctors when sharing personal health information and, as a result, are not concerned about data misuse.

“*My patients did not, per se, mention anything about this. They do trust their doctors where they are sending the information. So, I don’t think that they have this notion that their data would be used in some other way*” (P23).

Furthermore, participants expressed concerns about the lack of clear and comprehensive guidelines governing telemedicine practices. Many reported limited awareness of existing regulations and perceived significant risks to both patient care and provider safety, including incidents of video call scams and spam due to the exposure of personal contact details. They emphasized the need for standardized, specialty-specific guidelines and government-issued standard operating procedures (SOPs) to streamline telemedicine practices. Furthermore, participants highlighted the importance of secure consultation protocols such as recording consultations and provider training to enhance usability and awareness of these regulations.

“*Well, what are the exact guidelines? I’m not very well aware of what the exact guidelines are*” (P31).

### 3.4. Theme 3: Impact of COVID-19 on Non-Communicable Diseases

#### 3.4.1. New-Onset NCDs Following COVID-19 Infection

A rising number of patients, particularly young adults in their 30s to 40s, were found to develop non-communicable diseases, particularly diabetes, hypertension, and cardiovascular conditions, after recovering from COVID-19, even among those with no prior medical history or family history. Many healthcare providers attributed this trend, especially the onset of diabetes, to the use of corticosteroids during COVID-19 treatment. This pattern was not limited to adults; one respondent observed that neonates born to COVID-19-positive mothers who had received steroid treatment also exhibited elevated blood sugar levels shortly after birth.

“*There are a greater number of cases of non-communicable disease. We find blood pressure at an early age. We find heart involvement at an early age. We find lung damage at an early age. This thing normally occurs internationally after the age of 60 years in India. These occur after the age of 40 years. But even now because of post-COVID, we are seeing these cases after the age of 30 years*” (P02).

#### 3.4.2. Exacerbation of Pre-Existing NCDs Post-COVID

In addition to new-onset NCDs, healthcare providers observed an exacerbation of pre-existing NCDs following COVID-19. Patients with previously well-managed diabetes and hypertension experienced poor glycemic and blood pressure control, often necessitating increased dosages or additional medications. Providers also reported deterioration in chronic respiratory and cardiovascular conditions, accompanied by a rise in complications such as myocardial infarction, thromboembolic events, and stroke, many of which were increasingly seen in younger adults.

“*People are having respiratory problems; COPD is quite high. They are not getting relieved by whatever conventional method we have. Similarly, the hypertension is going up. Cardiovascular disease is also going up. The strokes are going up, and joint issues, the pain, and the weakness are also going up. Then we have cancers, which are also going up*” (P04).

#### 3.4.3. Continuity of Care in Managing NCDs During and After COVID-19

The pandemic strained healthcare delivery, particularly during peak waves, disrupting timely access to in-person care and essential resources. Telemedicine emerged as a vital tool for maintaining continuity of care, enabling remote consultations, especially in managing NCDs. Most healthcare providers reported that while the core treatment protocols for NCDs remained consistent, adjustments in doses, combination therapies, and symptomatic management were commonly employed based on disease progression and patient response. Providers from AYUSH systems noted a post-COVID shift in patient preference toward alternative treatments, particularly when conventional allopathic medications were less effective. Moreover, lifestyle interventions such as yoga, meditation, diet counseling, and holistic care also gained prominence as complementary approaches alongside standard treatment.

“*We are more focused on some lifestyle changes because we have realized that lifestyle interventions have had a lot of effect on the management of NCD patients. So, lifestyle changes, such as dietary habits, exercise, yoga, and meditation, have created such a positive change*” (P07).

### 3.5. Theme 4: Optimizing Digital Health and Telemedicine for NCD Care

Participants highlighted several forward-looking recommendations to enhance telemedicine delivery for NCD management, particularly in resource-limited settings. These recommendations emphasized system-level organization, capacity building, technological innovation, and the integration of telemedicine into preventive care.

#### 3.5.1. System-Level Organization and Workflow Integration

Participants recommended integrating telemedicine into routine clinical workflows through dedicated teleconsultation time slots to improve efficiency, reduce patient load, and enable telemedicine to function as a complementary service rather than an ad hoc solution.

“*Doctors should be trained in how to use this spectrum. Maybe if they have 8 or 10 h a day, we can have two hours only for teleconsultation so that the patient load that they are handling in OPDs as well as in patients can be avoided*” (P17).

#### 3.5.2. Capacity Building and Enhancing Awareness

Providers emphasized strengthening healthcare provider capacity through training in telemedicine use.

“*Doctors should be trained in how to use this spectrum*” (P17).

In addition, participants also stressed the need to simplify telemedicine platforms and increase public awareness through campaigns and education to encourage adoption.

“*The only thing I feel is awareness, like the government has launched many digital platforms, but people are not aware, and if they are aware, how will they use them? Increasing awareness, be it through social media or video or man-to-man, but it should be there for better utilization of resources*” (P30).

#### 3.5.3. Integration of Advanced Digital Technologies

Participants identified the integration of artificial intelligence (AI) and advanced digital tools as a promising approach to support real-time monitoring, triaging, and early identification of complications among NCD patients, thereby improving continuity of care and clinical decision-making.

“*The telemedicine role will increase in the future. With artificial intelligence coming in, we can improvise this telemedicine. Something like you can monitor a patient at different times in 1 h, 2 h, or while sitting in the hospital; with AI facilities, these things will be taken care of*” (P19).

#### 3.5.4. Telemedicine for Prevention and Health Promotion

Beyond disease management, participants emphasized the potential role of telemedicine in preventive care and health promotion, particularly among younger populations, by promoting healthy lifestyle modifications.

“*Making them aware about active lifestyles along with proper diet information or food habits can prevent them from getting NCDs in younger age groups, and this can be achieved through telemedicine*” (P20).

## 4. Discussion

This qualitative study aimed to elucidate healthcare providers’ perspectives on the use of telemedicine for NCDs management in India, particularly during and after the COVID-19 pandemic. The findings provide critical insights into both the opportunities and limitations of telemedicine within clinical settings, particularly from the perspectives of frontline providers operating across diverse medical systems.

The pandemic significantly altered the landscape of NCDs. Consistent with recent studies, participants in this study reported an increase in both new-onset and exacerbated cases of diabetes, hypertension, and cardiovascular conditions in the post-COVID period [19,20,21]. Several healthcare providers attributed the rise in diabetes cases to corticosteroid use during COVID-19 treatment, a link supported by emerging evidence from India and globally [22,23].

Furthermore, longitudinal data from Italy and Indonesia highlight similar post-pandemic trends, including a rise in new-onset hypertension and early-onset cardiac issues [24,25,26,27]. This alignment suggests that post-COVID NCD patterns in India are consistent with those reported internationally. Additionally, exacerbation of pre-existing NCDs following COVID-19 was widely observed, including worsening glycemic control and increased cardiovascular and respiratory complications. These findings are consistent with other studies indicating that COVID-19 may have accelerated chronic disease progression, potentially due to inflammation, stress, or long-term organ involvement [28,29]. These trends suggest a need to recognize COVID-19 not only as an acute respiratory illness but also as a trigger for long-term metabolic and vascular complications.

Telemedicine was widely recognized by providers as an essential tool for delivering follow-up care during lockdowns. The views expressed by healthcare providers in this study align with prior research demonstrating high levels of patient satisfaction, perceived convenience, and reduced travel time associated with telemedicine consultations [30,31,32]. Telemedicine also offers distinct advantages in chronic disease management by enabling remote monitoring, facilitating timely interventions, and supporting continuity of care through improved accessibility to healthcare services [33]. Furthermore, it has been shown to be a cost-effective approach for delivering long-term care [34]. Despite these advantages, providers reported several challenges that echo findings from previous studies, including the inability to conduct physical examinations, infrastructural limitations, and difficulties faced by elderly patients in using digital devices for telemedicine [35,36]. However, studies indicate that older adults, despite limited digital literacy, are generally receptive to digital health when supported by user-friendly platforms and adequate assistance [37,38]. This implies that barriers among older adults in using digital tools may be attributed more to limited support and non-user-friendly interfaces than to a lack of willingness to engage with technology. Additionally, providers highlighted that maintaining doctor–patient rapport was a key challenge, particularly in the absence of face-to-face interaction. Connectivity issues further disrupted continuity of care, often making it difficult for patients to reconnect with the same provider, ultimately impacting trust and the overall quality of care [35,39].

Providers from AYUSH systems observed a rising patient preference for alternative approaches, particularly AYUSH-based approaches and lifestyle-based interventions such as yoga, meditation, and dietary modifications, especially when conventional treatments failed to relieve persistent post-viral symptoms. This trend reflects India’s broader emphasis on preventive and holistic care in managing NCDs [40]. Incorporating such interventions into digital consultations may enhance chronic disease management and increase patient satisfaction, particularly among younger, urban populations seeking proactive and preventive healthcare solutions. Existing research supports the role of lifestyle medicine [41] and yoga-based interventions [42] in chronic disease prevention and early-stage NCD management, thereby contextualizing providers’ suggestions to explore integrating such approaches into digital consultations. Although specific to the Indian context, the emphasis on AYUSH systems parallels growing global interest in integrative and lifestyle-based approaches for chronic disease prevention and management.

Telemedicine has emerged as a promising long-term strategy for managing NCDs in India. However, its effective integration into routine healthcare delivery demands targeted system-level reforms. Healthcare providers recommended practical measures such as dedicating specific time slots for teleconsultations, simplifying digital interfaces, incorporating video-enabled consultations to enhance clinical assessment, and strengthening digital literacy among both patients and providers. These recommendations align with the objectives of India’s National Digital Health Mission (NDHM) and reflect the global transition toward hybrid models of care that integrate digital and face-to-face services for improved accessibility and continuity [43]. An important innovation highlighted by providers was the integration of artificial intelligence (AI) into telemedicine platforms. AI has the potential to support clinical triaging, issue early warnings, and facilitate continuous remote monitoring, particularly beneficial in overburdened or resource-limited healthcare settings. As AI technologies become more accessible, their application could significantly reduce outpatient workloads, improve chronic disease management, and enhance healthcare delivery in underserved areas.

### Limitations

This study draws on a purposive sample of 33 healthcare providers from diverse healthcare systems and settings. While their insights offer valuable depth, the findings may not be generalizable to all providers or regions. Additionally, the absence of patient perspectives limits understanding of telemedicine adoption from the user’s viewpoint.

A further limitation is the lack of longitudinal data, which restricts insights into how provider perceptions and the effectiveness of telemedicine may shift over time. Future research should include both provider and patient voices and employ mixed-method and longitudinal designs to evaluate the long-term impact, equity, and sustainability of digital health interventions in the post-pandemic landscape.

## 5. Conclusions

This study highlights the pivotal role of telemedicine in sustaining NCD care during the COVID-19 pandemic in India. Healthcare providers recognized several advantages of telemedicine, including enhanced accessibility, cost-effectiveness, and convenience, especially in underserved and high-risk settings. Despite these benefits, significant barriers persist, such as low digital literacy, inadequate infrastructure, and clinical challenges in managing complex cases remotely.

For telemedicine to be effectively integrated into long-term NCD care, these systemic and technological gaps must be proactively addressed. As India continues to face a growing NCD burden in the post-pandemic era, a coordinated strategy that combines digital innovation with patient-centered approaches, comprehensive provider training, and robust health system reforms will be critical to ensuring equitable and sustainable care delivery.

## Figures and Tables

**Table 1:** Socio-demographics of participants.

Health Care Providers (*n* = 33)	*n*	*n (%)*
**Age Group (Years)**		
25–34	10	30.30%
35–44	12	36.36%
44–54	7	21.21%
>55 years	4	12.12%
**Gender**		
Female	17	51.51%
Male	16	48.48%
**Education level**		
MBBS, MD/MS	17	51.51%
BDS, MDS	1	3.03%
BAMS, MD	4	12.12%
BHMS, MD	5	15.15%
BUMS, MD	1	3.03%
BSMS, Ph.D	2	6.06%
Pharmacist	1	3.03%
MPH, Ph.D	2	6.06%
**Health care settings**		
Government hospitals	22	66.66%
Private Hospitals	3	9.09%
PHCs	1	3.03%
Private Clinics	5	15.15%
Academics	2	6.06%
**Working experience in (years)**		
1–10	10	30.30%
11–20	15	45.45%
>21	8	24.24%

## Data Availability

Data used in the study will be provided by the corresponding author upon reasonable request.

## Supplementary Materials

The following supporting information can be downloaded at https://www.mdpi.com/article/10.3390/ijerph23020203/s1. Table S1: Consolidated criteria for reporting qualitative studies (COREQ): 32-item checklist; Table S2: Semi-structured interview guide; Table S3: Coding tree. Ref. [44] are cited in Supplementary Materials.

## Abbreviations

MBBS	Bachelor of Medicine and Bachelor of Surgery
MD	Doctor of Medicine
BDS	Bachelor of Dental Surgery
MDS	Master of Dental Surgery
BAMS	Bachelor of Ayurvedic Medicine & Surgery
BHMS	Bachelor of Homeopathic Medicine & Surgery
BUMS	Bachelor of Unani Medicine & Surgery
BSMS	Bachelor of Siddha Medicine & Surgery
Ph.D.	Doctor of Philosophy
MPH	Master in Public Health
PHCs	Primary Health Centre
AYUSH	**A**yurveda, **Y**oga & Naturopathy, **U**nani, **S**iddha, and **H**omeopathy
WHO	World Health Organization
NCD	Non-communicable diseases
NDHM	National Digital Health Mission
COREQ	Consolidated Criteria for Reporting Qualitative Research
ABDM	Ayushman Bharat Digital Mission
