# Supplementary material for: Healthcare Providers’ Perspectives on Telemedicine for NCD Management During and After COVID-19 in India: A Qualitative Study
Source: Int J Environ Res Public Health. 2026 Feb 5;23(2):203. doi: 10.3390/ijerph23020203 (PMC12940190; doi:10.3390/ijerph23020203)
Supplement: Supplementary file 1 [file ijerph-23-00203-s001.zip › ijerph-4063273-supplementary.pdf]

Table S1. Consolidated criteria for reporting qualitative studies (COREQ): 32-item checklist.

**Domain 1: Research team and reflexivity**			
**Personal Characteristics**			
1.	Interviewer/facilitator	Which author/s conducted the interview or focus group?	Page 4
2.	Credentials	What were the researcher's credentials? E.g. PhD, MD	Page 4
3.	Occupation	What was their occupation at the time of the study?	Page 4
4.	Gender	Was the researcher male or female?	Page 4
5.	Experience and training	What experience or training did the researcher have?	Page 4
**Relationship with participants**			
6.	Relationship established	Was a relationship established prior to study commencement?	Page 4
7.	Participant knowledge of the interviewer	What did the participants know about the researcher? e.g. personal goals, reasons for doing the research	All reasons were disclaimed (doctoral interests)
8.	Interviewer characteristics	What characteristics were reported about the interviewer/facilitator? e.g. Bias, assumptions, reasons and interests in the research topic	IR's interest in the research topic (doctoral interests) was disclaimed to all participants.
**Domain 2: study design**			
**Theoretical framework**			
9.	Methodological orientation and Theory	What methodological orientation was stated to underpin the study? e.g. grounded theory, discourse analysis, ethnography, phenomenology, content analysis	Page 4 -5
**Participant selection**			
10.	Sampling	How were participants selected? e.g. purposive,	Page 3

		convenience, consecutive, snowball	
11.	Method of approach	How were participants approached? e.g. face-to-face, telephone, mail, email	Page 3
12.	Sample size	How many participants were in the study?	Page 3
13.	Non-participation	How many people refused to participate or dropped out? Reasons?	Page 3
**Setting**			
14.	Setting of data collection	Where was the data collected? e.g. home, clinic, workplace	Page 3
15.	Presence of non-participants	Was anyone else present besides the participants and researchers?	No other individuals were present
16.	Description of sample	What are the important characteristics of the sample? e.g. demographic data, date	Table S1
**Data collection**			
17.	Interview guide	Were questions, prompts, guides provided by the authors? Was it pilot tested?	Page 4
18.	Repeat interviews	Were repeat interviews carried out? If yes, how many?	No repeated Interviews were conducted
19.	Audio/visual recording	Did the research use audio or visual recording to collect the data?	Page 3
20.	Field note	Were field notes made during and/or after the interview or focus group?	Some filed notes were made during the interviews
21.	Duration	What was the duration of the interviews or focus group?	Page 3
22.	Data saturation	Was data saturation discussed?	Page 3
23.	Transcripts returned	Were transcripts returned to participants for comment and/or correction?	Page 5
**Domain 3: analysis and findings**			
**Data analysis**			

24.	Number of data coders	How many data coders coded the data?	Page 4
25.	Description of the coding tree	Did authors provide a description of the coding tree?	Yes-See S3 Appendix (code book)
26.	Derivation of theme	Were themes identified in advance or derived from the data?	Both
27.	Software	What software, if applicable, was used to manage the data?	Page 4
28.	Participant checking	Did participants provide feedback on the findings?	No
**Reporting**			
29.	Quotations presented	Were participant quotations presented to illustrate the themes/findings? Was each quotation identified? e.g. participant number	Yes
30.	Data and findings consistent	Is there consistency between the data presented and the findings?	Yes
31.	Clarity of major themes	Were major themes clearly presented in the findings?	Yes
32.	Clarity of minor themes	Is there a description of diverse cases or discussion of minor themes?	Page 6

Table S2: Semi-structured interview guide

Introduction

Good morning!

My name is Dr. Isha Rathi, and we are conducting a study on the management of non-communicable diseases, with a focus on the adoption of digital consultations during the COVID-19 pandemic. The purpose of this study is to explore the challenges, benefits, and future potential of telemedicine in managing non-communicable diseases (NCDs) in India from the perspective of healthcare providers, both during and after the pandemic. Thank you for agreeing to participate and for taking the time to speak with us today.

In this conversation, we would like to hear about your experiences and viewpoints. Your input is important to us, and we encourage you to express your thoughts freely. With your permission, we will record this interview and also take notes to assist with our analysis. If at any time you would prefer not to be recorded, please let us know. All information you share will remain confidential and will be used solely by our research team for the purposes of this study.

The interview will take approximately 20-25 minutes.

**Interview Process:**<br><br>Written informed consent was taken prior the start of interview.<br><br>Just to inform you, this conversation is being recorded.	
	**Questions**
**Telemedicine benefits, challenges and satisfaction**	1. Could you please share your experiences with using telemedicine during and after the pandemic?
	2. What are the challenges in using telemedicine?
	3. If you were to rate telemedicine on a scale of 1 to 10 for its effectiveness in healthcare delivery, how would you rate it?
	4. Is there anything you'd like to share that could help us better understand the future potential of telemedicine?
**Impact of COVID-19 on Non-Communicable Diseases**	5. In your experience, how has the use of telemedicine influenced the management of NCD's ?
	6. What is the impact of COVID-19 on NCD?
**Optimizing Digital Health and Telemedicine for NCD Care**	7. What are your thoughts on use of digital health for NCD care?

Thank you for your time; RECORDING STOP

Table S3. Coding tree

Themes	Sub themes
**Theme 1:** Perceived Benefits and Satisfaction with Telemedicine	1.1. Access, Affordability, and Time–Cost Efficiency<br>1.2. Continuity of Care During the COVID-19<br>1.3. Psychological Reassurance and Perceived Support<br>1.4. Provider Satisfaction with Telemedicine
**Theme 2:** Challenges in Telemedicine Use	2.1 Clinical Limitations<br>2.2 Technological and Infrastructure Barriers<br>2.3 Digital Literacy and Heterogenous awareness<br>2.4 System-Level and Scheduling Constraints<br>2.5 Data Safety, Privacy, and Regulatory Concerns
**Theme 3:** Impact of COVID-19 on Non-Communicable Diseases	3.1 New-Onset NCDs following COVID-19 Infection<br>3.2 Exacerbation of Pre-existing NCDs post-COVID<br>3.3 Continuity of Care in Managing NCDs During and after COVID-19
**Theme 4:** Optimizing Digital Health and Telemedicine for NCD Care	4.1 System-Level Organization and Workflow Integration<br>4.2 Capacity Building and Enhancing Awareness<br>4.3 Integration of Advanced Digital Technologies<br>4.4 Telemedicine for Prevention and Health Promotion

Reference

1. Tong A, Sainsbury P, Craig J. Consolidated criteria for reporting qualitative research (COREQ): a 32-item checklist for interviews and focus groups. Int J Qual Heal Care. 2007;19(6):349-357.
